# Productive and Physico-Chemical Parameters of Tomato Fruits Submitted to Fertigation Doses with Water Treated with Very Low-Frequency Electromagnetic Resonance Fields

**DOI:** 10.3390/plants11121587

**Published:** 2022-06-16

**Authors:** Fernando Ferrari Putti, Bianca Bueno Nogueira, Angela Vacaro de Souza, Eduardo Festozo Vicente, Willian Aparecido Leoti Zanetti, Diogo de Lucca Sartori, Jéssica Pigatto de Queiroz Barcelos

**Affiliations:** 1 School of Sciences and Engineering, São Paulo State University (UNESP), Rua Domingos da Costa Lopes 780, Tupã 17602-496, SP, Brazil; bianca.nogueira@ufv.br (B.B.N.); angela.souza@unesp.br (A.V.d.S.); eduardo.vicente@unesp.br (E.F.V.); willian.zanetti@unesp.br (W.A.L.Z.); diogo.sartori@unesp.br (D.d.L.S.); 2 School of Agriculture, São Paulo State University (Unesp), Rua José Barbosa de Barros 1870, Botucatu 18610-307, SP, Brazil; jessica.barcelos@unesp.br

**Keywords:** *Solanum lycopersicum* (L.), post-harvest quality, water electro magnetization, minerals quantification, fertigation

## Abstract

It is known that poorly performed fertigation directly impacts on tomato production and biometric components. In addition, consumers are also affected by interrelated characteristics that interfere with the acceptability of the fruit, such as the physicochemical parameters and nutrients in the fruit. Thus, eco-friendly technologies, such as irrigation with ultra-low frequency electromagnetic treated-water, which attenuates the inadequate management of fertigation, are essential to improve marketable fruit yields. Thus, the objective of the present work was to investigate the impact of treated water with very low-frequency electromagnetic resonance fields in physical, chemical and nutritional parameters at different nutrient solution strengths in tomato fruits. In this study, experiments were carried out in randomized blocks and five doses of fertigation were used (1.5; 2.5; 4.0; 5.5; and 7.0 dS m^−1^), employing two types of water: electromagnetically treated and untreated. It can be seen that the fertigation affected some parameters, mainly the number of fruits with blossom-end rot, fruit size, and weight. Variance analysis (ANOVA) was performed with the subsequent use of the Tukey test. In all statistical tests, a confidence level of 95% was considered. The soluble solids content increased by 28% as a function of the fertigation doses. The electromagnetically treated water reduced the number of fruits with blossom-end rot by 35% (*p* < 0.05). Overall, electromagnetic water improved the physicochemical quality parameters and the nutritional status of tomato fruits. Thus, this study demonstrated that green technology could leverage tomato fruit production and quality.

## 1. Introduction

Tomato (*Solanum lycopersicum* L.) fruits are among the most widely consumed vegetables worldwide, placing at the 10th most produced vegetable crop and the 11th most produced crop in 2017, in the world [1,2]. It can be consumed fresh or in different processed forms (i.e., preserves, jams, etc.).

The tomato stands out as one of the leading culinary vegetables consumed by the world’s population. The fruit is characterized by having low calories and fats, besides basically containing water, sugar (glucose and fructose), acids (acetic, lactic and malic acids), vitamin C, pro-vitamin A (β-carotene), and also traces of potassium, phosphorus and iron [3]. For the good agronomic performance of the tomato crop, its cultivation has been carried out in a protected environment to reduce climatic impacts and pest attacks [4]. Associated with this cultivation in greenhouses, irrigation management is an efficient technique to optimize tomato production [5].

It is necessary to develop technologies aimed at the efficient management of water resources and fertilizers since, in protected environments, one of the most common negative factors is the ionic strength of a nutrient solution, with the occurrence of salt accumulation due to the absence of leaching and/or the application of high fertilizer doses. Therefore, it is essential to use technologies that mitigate the ionic strength to guarantee the final product quality [6].

The tomato is characterized by a plant that is moderately sensitive to salinity. It is crucial to highlight that several studies found different responses regarding the effects of the salinity of the nutrient solution on the physico-chemical characteristics of the fruits, with a maximum tolerance of 2.5 dS m^−1^. At higher levels of nutrient solution salinity (above 2.5 dS m^−1^), there is a reduction in productivity, nutritional imbalance, and an increase in ROS production, which can lead to the plant death [7,8].

To reduce the effects of salinity of the nutrient solution, current research has been demonstrating the beneficial effects of the electromagnetic treatment of irrigation water [9]. In a study using electromagnetically treated water in salinized soils, it was found that the effects of a high salt concentration were reduced, providing less stress to the plant [10]. Regarding the impact caused by water deficit stress, it was demonstrated that magnetic water treatment could increase the physico-chemical contents of tomato fruits [11].

Studies have demonstrated that, when water is induced into a magnetic or electromagnetic field, there are changes in their molecular interactions, in which there may be variations in the calcium carbonate crystal structure [11] and changes in the distribution of salts between the soil layers, reducing its content in the upper layers [12]. In addition, studies have already verified differences in water characteristics such as viscosity, surface tension, light refraction index, electrical conductivity, and light absorption capacity [13], as well as changes in soil characteristics such as moisture and moisture reduction in the applied water volume in irrigation. Therefore, the importance of developing studies that shed light on the behavior of water when exposed to magnetic and electromagnetic treatments is remarkable, as studies on these topics are very scarce, especially those involving soil salinization [14]. Finally, the objective of the present work was to evaluate the physicochemical parameters under the effect of electromagnetically treated water at nutrient solution strengths in the tomato crop.

## 2. Materials and Methods

### 2.1. Experimental Area

The experimental stage of this research was developed in the Experimental Area of the School of Sciences and Engineering, Campus de Tupã, São Paulo, Brazil, with a geographical location defined by the coordinates 22°51′ S and 48°26′ W and an average altitude of 786 m above the sea level. The average monthly wind speed at 10 m high is 3.1 ms^−1^ and the average daily global solar energy is 4772.13 Wh m^−2^. The protected environment is a tunnel type, with dimensions of 25 m in length, 6 m in width, and height on the sides of 3 m and the center of 5 m. The cover is made with transparent polyethylene film, with 150 μm of thickness. The sides are made of ‘sombrite’ canvas, with 30% shading, aiming at the interception of insects and animals. The greenhouse is located along its length in the north/south direction. The experiment was carried out in the southern hemisphere winter, from June to September 2018.

### 2.2. Treatments

The experimental design adopted was randomized blocks, in a 2 × 5 factorial scheme, composed of two factors: two types of water (ETW = water treated with very low-frequency electromagnetic resonance fields and UTW = untreated water) and five strength levels of nutrient solution (1.5; 2.5; 4.0; 5.5; and 7.0 dS m^−1^) for tomato culture. Thus, the experiment consisted of ten treatments with four replications, totaling 40 parcels. Treatments were arranged in a pot of 8 dm^3^ (one plant per pot), filled with coconut fiber substrate. The pots were allocated in five rows, with a spacing of 0.35 m between plants and 1 m between rows.

### 2.3. Planting and Plant Management

The cultivar used in the experiment was HS 1188 by Horticeres Seeds, São Paulo, Brazil. Sowing was carried out in plastic trays of 160 cells and a mixture based on pine bark and coconut fiber. Bioplant^®^ was used as the substrate, with three seeds per cell sown, which, after germination, were submitted to thinning, leaving only one seedling per cell. Seedling transplantation occurred 35 days after sowing (DAS), with seedlings transplanted to pots of 8 dm^3^ (one plant per pot), filled with coconut fiber. After planting and the consequent crop development, the lateral buds were eliminated to maintain only one main branch up to approximately 2 m in height, which was spawned, paralyzing the vertical development. In addition, the tutoring of tomatoes was carried out with the aid of plastic tape, supported by a wire installed above and in the direction of the cultivation lines.

### 2.4. Electromagnetic Treatment of Water

The equipment used for the electromagnetic treatment of water is Aqua-4D, which performs the physical treatment of the water. Quantum physics indicates that water is an organized and structured matter, not chaotic, as one might think. Water is an element that can adapt to very different structures. Thus, when the water passes through an electromagnetic field, it better dissolves and distributes minerals in a natural system. In this way, it causes increased water retention in the soil and better absorption of minerals by the plants. Thus, the equipment consists of a pre-programmed electronic panel to generate electromagnetic signals and a tube that the water passes through, where it receives the signal. The affected water after treatment has effects for up to 15 h of magnetic field tracking. The magnetic field fluctuation recorded is relatively low with B_max_ =  0.023 mT and B_min_  =  0.008 mT, with B_avg_  =  0.0155 mT for magnetic water, while for tap water, the estimated the values werer B_max_ = 0.0185 mT and B_min_  =  0.005 mT, with B_avg_  =  0.01175 mT [12,15].

### 2.5. Irrigation Management

Irrigation was performed using a localized system, and approximately every 20 min the plan was activated and remained only for two minutes. The operation was between 8:00 am and 5:00 pm, totaling 108 pulses, which had a total duration of 216 min irrigating time. It is important to mention that the system did not activate at night.

The installed drippers had a flow rate of 3.6 L h^−1^; in this format, an average of 12.96 L was irrigated per day. The metering pump could inject 10 L h^−1^ of solution into the irrigation system. It is emphasized here that this solution was concentrated and the irrigation solution was adjusted to 1.5; 2.5; 4.0; 5.5; and 7.0 dS m^−1^. Therefore, five reservoirs were adopted where the solutions were stored in a concentrated manner. When the irrigation system was activated, fertilizers were injected.

Table 1 presents the fertilizers used and their respective amounts for the fertigation rates, applied throughout the experiment. Two periods of fertigation were adopted, the first being the plant development phase and the second when the first fruits appeared, and the fertilization was carried out according to [16,17].

### 2.6. Physico-Chemical Analysis of the Fruits

#### 2.6.1. Soluble Solids (SS)

Total soluble solids were determined using a direct measurement employing an Optech RMT model refractometer. The results were expressed in BRIX.

#### 2.6.2. Titratable Acidity (TA)

Titratable acidity was expressed in grams of citric acid per 100 g of pulp (g of citric acid 100 g^−1^). The quantification is obtained by titrating 5 g of homogenized and diluted pulp to 100 mL of distilled water, with standardized 0.1 N sodium hydroxide solution, with phenolphthalein used as the indicator [18,19].

#### 2.6.3. Ratio SS/TA

This is the ratio between the content of soluble solids and titratable acidity. The results of this analysis are dimensionless.

#### 2.6.4. pH

The pH was measured in the crushed pulp of the fruits using a potentiometer (Digital DMPH-2, Lucadema, São Paulo, Brazil) [19].

### 2.7. Distance between Internodes

The distance between internodes, expressed in centimeters (cm), is the distance between all the plant nodes, located from the beginning of the bifurcation of the stems to the first leaf just below the last one. This variable was measured using a tape measure.

### 2.8. Branch Weight

The branch weight was carried out on a scale, with data expressed in grams (g).

### 2.9. Average Length and Fruit Diameter

The average length and the diameter of the fruit were determined in cm, with the aid of a caliper in the middle region of the fruit.

### 2.10. Nutritional Analysis

To assess the nutritional status of the fruits, four fruits were collected per parcel, and only fruits in the maturity stage were collected. The samples were dried to a constant weight and then ground and digested in nitric-perchloric solution. The levels of P, K, Ca, S, Mg, B, Cu, Fe, Mn, Mo, Ni and Zn were determined using an ICP-MS equipment. The total N content was determined by sulfuric digestion and by default a Kjeldhal distillation method, described elsewhere [17].

### 2.11. Statistical Analysis

The analysis of variance (ANOVA) was performed with the subsequent use of the Tukey test; when there was an effect on nutrient solution strengths, regression analysis was performed. In this case, for the research that showed an interaction between the factors (types of water and conductivity levels), they were compared between averages, first reaching the levels of one of the factors in each of the levels of the other factor, and vice-versa. When there is no interaction between the factors, comparisons were made between the averages only for the factor levels that showed a significant difference in ANOVA. In all statistical tests, a confidence level of 95% was considered [20]. The R software was used to apply the described tests.

## 3. Results

Table 2 presents the *p*-values of the analysis of variance. Thus, it first presented the interactions of the effects; when isolated effects were also significant, it was not presented. It sought to discuss the effect of the exchange, but when there was no significance, it presented the isolated effects.

### Biometric Analysis

**Figure 1:**
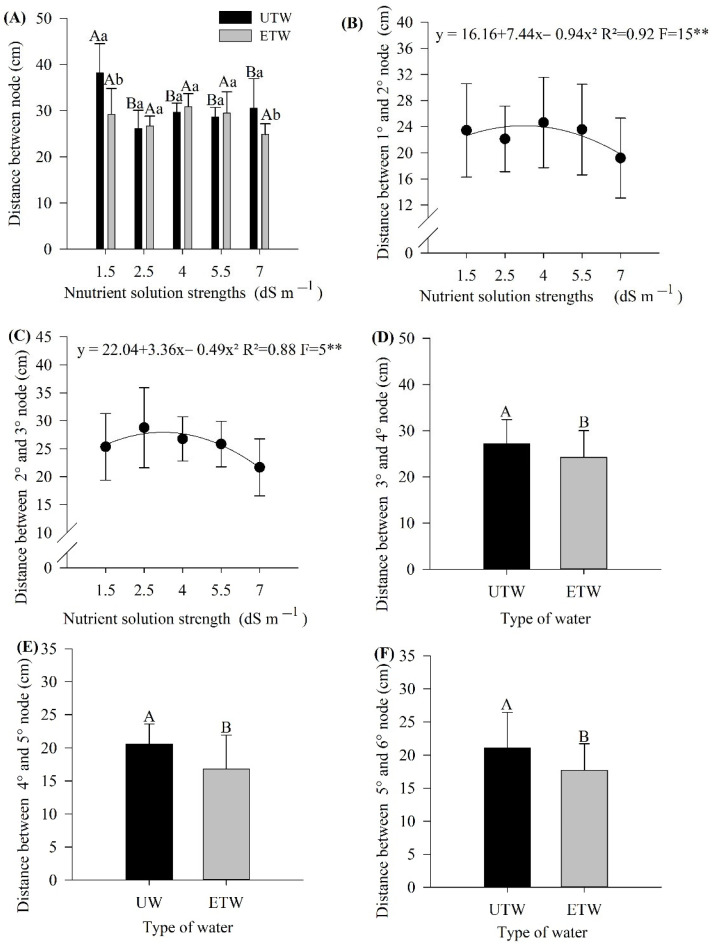
Distance between nodes in tomato culture submitted to different strength nutrient solution with electromagnetically treated water (ETW) and untreated water (UTW). (**A**) Distance between node (cm), (**B**) Distance between 1° and 2° node (cm), (**C**) Distance between 2° and 3° node (cm), (**D**) Distance between 3° and 4° node (cm), (**E**) Distance between 4° and 5° node (cm), (**F**) Distance between 5° and 6° node (cm) According to the Tukey test, the means followed by the same letter, lower case comparing the type of water and upper case comparing the strength of the nutrient solution, do not differ statistically (*p* < 0.05). The error bars indicate the standard deviation of the average of four repetitions (*n* = 4). ns: not significant, ** significant at 1%.

The internode branch distance is an indirect way of determining the efficiency of the plant, since the reduction in internodes means that the plant does not have energy expenditure for its maintenance. In the present study, six internodes were analyzed, so it was possible to verify that, for the first internode, there was an interaction of nutrient solution strength and applied water (Figure 1A) for the strengths of 1.5 and 7.0 dS m^−1^. In this case, the internode distance was greater in tomatoes submitted to irrigation with ETW. When analyzing only the effect of the irrigation water, it was noted that irrigation with UTW showed a difference only for the strength of the nutrient solution of 1.5 dS m^−1^, with a greater internode distance. In contrast, for ETW, no difference was found for this parameter.

Regarding the distances between the first and second internodes and the second and third internodes, there was only the effect on the strength of the nutrient solution (Figure 1B,C). The maximum point for the quadratic adjustment occurred close to the strength of the nutrient solution of 2.5 dS m^−1,^ followed by a significant reduction, for both distances (Figure 1B,C). Lower internode distances were observed for plants that received ETW (*p* < 0.05) (Figure 1D,E,F respectively).

**Figure 2:**
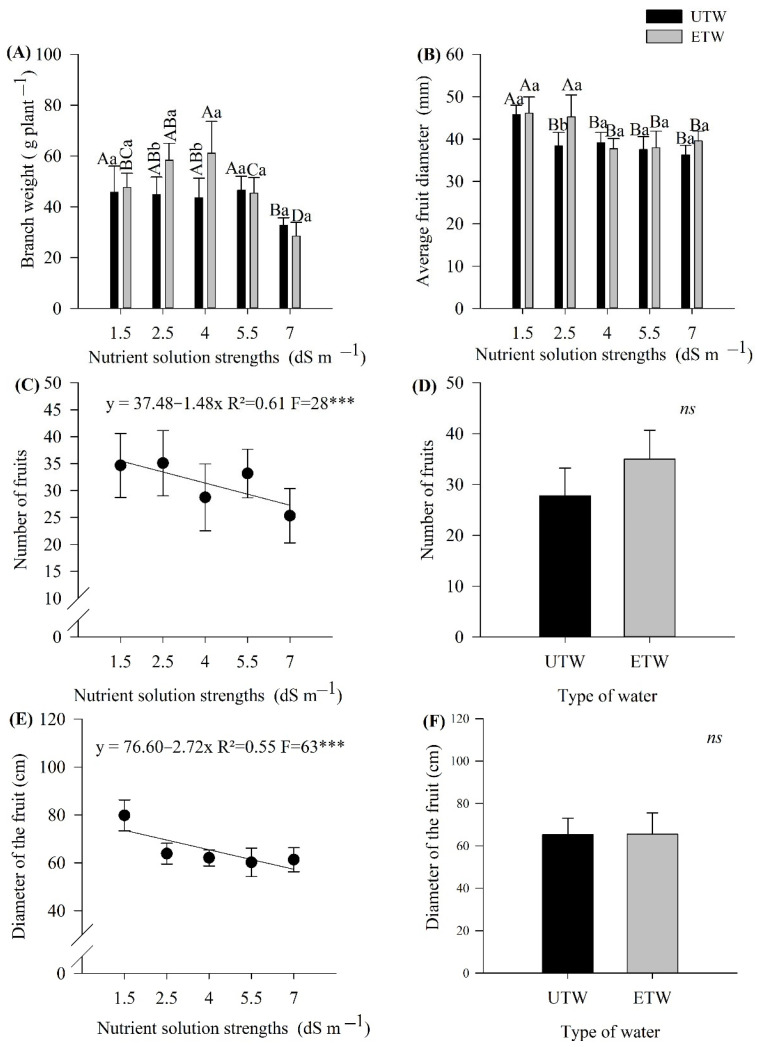
Biometric parameters of the tomato crop fruits were subjected to different strength nutrient solution with electromagnetically treated water (ETW) and untreated water (UTW) (**A**) Branch Weigth, (**B**) Average fruits diameter, (**C**,**D**) Number of fruits, (**E**,**F**) Diameter of fruits. According to the Tukey test, the means followed by the same letter, lower case comparing the type of water and upper case comparing nutrient solution strength do not differ statistically (*p* < 0.05). The error bars indicate the standard deviation of the average of four repetitions (*n* = 4). ns: not significant, *** significant at 1%.

The biometric parameters of the tomato crop showed a significant effect depending on the strength of the nutrient solution and the type of water. An interaction was observed between the factors (Figure 2A). The nutrient solution strengths of 2.5 and 4.0 dS m^−1^, irrigated with ETW, presented greater branch weight. However, the lowest branch weight was observed with the nutrient solution strength of 7.0 dS m^−1^ for both types of water (Figure 2A).

The average fruit diameter was significantly affected by nutrient solution strength and the type of water used. For the of 2.5 dS m^−1^ strength of the nutrient solution, there was a larger diameter when irrigated with ETW. When analyzing the interaction between the nutrient solution strengths of 1.5 and 2.5 dS m^−1^, it can be inferred that they provided the largest fruit diameter (Figure 2B).

Changes in fruit length and the number of fruits occurred only due to the nutrient solution strength and not the type of water. The increase in the nutrient solution strength produced a decrease in the fruit diameter, in which the fruit reduced its size by 27% comparing the 1.5 dS m^−1^ nutrient solution strength to the 7 dS m^−1^ (Figure 2C,D). The number of fruits also showed a linear reducted in which the increase in concentration reduction in the number of fruits, and each 1.5 dS m^−1^ increase in strength lead to a 15% reduction in the number of fruits. (Figure 1D,E).

**Figure 3:**
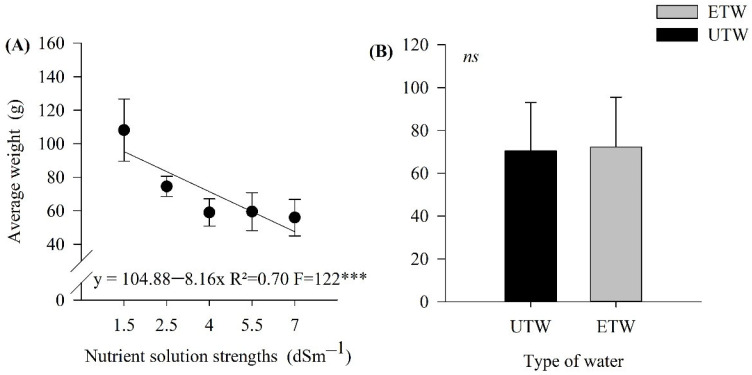
The average weight of the tomato crop fruits submitted to different strength nutrient solution with electromagnetically treated water (ETW) and untreated water (UTW), (**A**) effect only of the different strength nutrient solution, (**B**) effect of the type of water used in irrigation. The error bars indicate the standard deviation of the average of four repetitions (*n* = 4). ns: not significant, *** significant at 1%.

Regarding the production, the average fruit weight showed an effect only as a function of the nutrient solution strength. There was a linear decreasing effect due to the increase in the fertigation rates. It can be observed that there was a reduction of approximately 50% in weight for the nutrient solution strength of 1.5 dS m^−1^, in comparison with to the 7.0 dS m^−1^ solution. There was no effect comparing the type of water treatment adopted (Figure 3).

**Figure 4:**
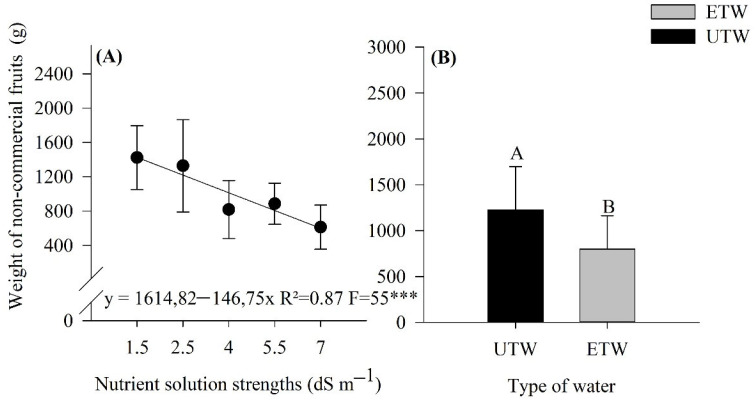
Weight of non-commercial fruits (with apical rot) from the tomato crop submitted to different strenght nutrient solution with electromagnetically treated water (ETW) and untreated water UTW). (**A**) effect only of the different strength nutrient solution, (**B**) effect of the type of water used in irrigation. According to the Tukey test, the means followed by the same letter, lower case comparing the type of water, do not differ statistically (*p* < 0.05). The error bars indicate the standard deviation of the average of four repetitions (*n* = 4). *** significant at 1%.

Another important factor in the production of tomatoes, which causes enormous damages for producers, is apical rot, popularly known as blossom-end rot, since it decreases the number of commercial fruits. This factor is directly related to the availability of calcium, fruit resistance, or water stress. Figure 4 shows the weight of non-commercial fruits, apical rot, and the interaction between the factors under study, nutrient solution strength, and irrigation water. Thus, it was found that there was no interaction between the factors but only the effect of the isolated factors, in which the nutrient solution strengths caused a linear decreasing impact as the strength increased, resulting in approximately a 50% reduction in the weight of non-commercial fruits, when the nutrient solution strength goes from 1.5 to 7.0 dS m^−1^. As for the type of water, it was found that a greater weight of fruits had a blossom-end rot when irrigated with untreated water, which differed significantly when the fruits were washed with electromagnetically treated water, with a difference of approximately 30%.

**Figure 5:**
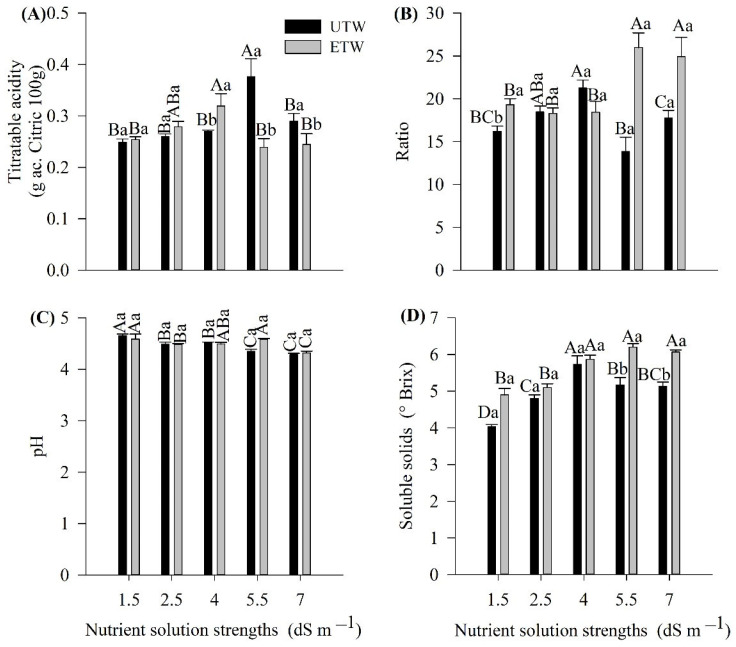
Physico-chemical characterization of the fruits of tomato cultivation subjected to different strength nutrient solution with electromagnetically treated water (ETW) and untreated water (UTW). (**A**) Tritratable Acidity, (**B**) Ratio, (**C**) pH and (**D**) Soluble solids. According to the Tukey test, the means followed by the same letter, lower case comparing the type of water, does not differ statistically (*p* < 0.05). The error bars indicate the standard deviation of the average of four repetitions (*n* = 4).

The physico-chemical parameters of the fruit were affected by the nutrient solution strengths and the type of water (Figure 5). The titratable acidity showed a higher concentration for the nutrient solution strength of 5.5 dS m^−1^, when under irrigation with ETW and for the nutrient solution strength of 7.0 dS m^−1^, when irrigated with UTW (Figure 5A). Compared to the 4.0 dS m^−1^ nutrient solution strength, a greater concentration occurred when irrigated with ETW (*p* < 0.05) and the 5.5 and 7.0 dS m^−1^ strengths, when irrigated with UTW.

The ratio showed an interaction between the factors. The nutrient solution strengths of 5.5 and 7.0 dS m^−1^ showed higher values when irrigated with ETW and differed significantly when the irrigation with UTW was adopted (Figure 5B). When irrigated with UTW, it was found that the nutrient solution strength of 4.0 dS m^−1^ showed the highest value among the nutrient solution strengths applied.

The pH of the fruits ranged from 4.0 to 5.5. The irrigation with ETW presented higher values for the doses 1.5 and 5.5 dS m^−1^. In addition, when the irrigating with UTW, the most elevated pH occurred with the dose of 1.5 dS m^−1^ (Figure 5C). It was also found that there was an interaction between the factors, in that, as the strength of the nutrient solution increased, there was a reduction in the pH values. The nutrient solution strength reduced the fruit pH in both irrigation systems (Figure 5C).

The content of soluble solids was raised as the nutrient solution strengths increased. However, it did not show a correlation with the potassium content. The content of soluble solids showed a significant increase, due to the increase in the nutrient solution strengths (Figure 5D).

It can be seen from the results obtained that, for tomato fruits irrigated with ETW, there was an increase in the concentration of phosphorus (P), calcium (Ca), magnesium (Mg), sulfur (S), boron (B) and manganese (Mn). Interestingly, the zinc (Zn) element had the opposite effect. In neutrality, the elements nitrogen (N), potassium (K), iron (Fe), and copper (Cu) showed no significant difference for the types of water employed. In addition, generally, it was possible to verify a linear and quadratic effect for nutrients as the nutrient solution strengths were increased (Table 3).

The nitrogen (N) content showed an increasing linear effect as a function of fertigation rates. It should be noted that there was no difference depending on the type of water (Table 3).

The phosphorus (P) content showed an interaction between water type factors and nutrient solution strengths. For the nutrient solution strength of 2.5 dS m^−1^, under irrigation with ETW, it differed significantly from irrigation with UTW. Plants under irrigation with ETW had the highest content of the element for the nutrient solution strength of 2.5 dS m^−1^ and when under irrigation with UTW, the highest content was found for the 5.5 dS m^−1^ solution. When checking the type of water in isolation, it was observed that the highest content of the element occurred under ETW irrigation.

The potassium (K) content did not interact with the factors studied of, water type, and nutrient solution strength. The effect was noticed only for the nutrient solution strengths, in which the linear effect was incremented as the nutrient solution strengths increased.

The calcium (Ca) content showed an interaction between the factors; that is, the results differed significantly for the types of water. The irrigation with ETW exhibited a higher content for the element. When the nutrient solution strength effect was observed, irrigation with ETW showed a higher calcium content for the nutrient solution strength of 1.5 dS m^−1^, and for ETW, the highest levels, which did not differ significantly, were for the 2.5, 4.0, and 5.5 dS m^−1^ strength It should be noted that, for the 1.5 and 7.0 dS m^−1^ solutions, the highest levels were observed for treatment with ETW.

For magnesium (Mg), the interaction between factors was found. The highest levels of the element, when irrigated with ETW and UTW, were given by the 2.5 dS m^−1^ and 5.5 dS m^−1^ nutrition solution strengths, respectively. When analyzing the effect of the water type in isolation, it was found that there was a higher content when irrigated with ETW.

The sulfur (S) content did not show any interaction between the factors, but the water type adopted affected it significantly. Thus, ETW irrigation showed the highest levels of this element. For the boron (B) content, there was an interaction between the factors, in and the nutrient solution strength of 2.5 dS m^−1^, when irrigated with ETW, showed the largest increment (*p* < 0.05).

The manganese (Mn) element showed an interaction, since the ETW showed a positive effect and increased absorption, in general, within each dose, with decreasing linear effects. Only for the 4.0 dS m^−1^ and 5.5 dSm^−1^ nutrition solution strengths the UTW demonstrated a positive effect. The iron content did not affect the nutrient solution strength and water type. It was observed did the 1.5, 4.0 and 5.5 dS m^−1^ solutions presented a higher content of this element, when irrigated with ETW.

The copper (Cu) content showed an interaction between the factors. Thus, the 1.5 and 5.5 dS m^−1^ nutrient solution strengths showed a higher concentration of this metal when under irrigation with ETW. The other nutrient solution strengths did not show statistical differences. Finally, for the zinc (Zn) content, it was found that there was an interaction. In general, it was found that higher concentrations of this metal occurred for treatments that were irrigated with UTW.

## 4. Discussion

Internode length plays an important role for the improvement of plant architecture in tomato plants [21]. In studies with tomato culture, the distance between the tomato internodes is a parameter that can indicate the potential culture productivity, since it is directly related to yield [22,23]. Several factors are related, such as temperature, genotypes [24] and density [22,25]; it has been show that, in management of pepper cultivation with higher nitrogen doses, through fertigation, caused stem reduction. Thus, these data corroborate with the results obtained in the present study. It is important to highlight that, after the third internode, the observed sizes were larger when irrigated with untreated water, that is, the electromagnetic water provided the plants with less energy expenditure, since, the internode distance was shorter, in general, which may have contributed to the positive increase in other parameters. In addition, it should be emphasized that the distance between internodes directly impacted the number of fruits per plant. Furthermore, for [26], the internode distance and the number of fruits per plant, plant height and fruit weight are variables directly related to the yield and fruit quality. The internode distance is directly associated with the abiotic factors that provide oxidative stress in plants [27,28]. In the present article, this was verified, as the type of water applied attenuated the stress effects, and from the 3rd point onwards, only the impact of the type of water was observed. In this way, the hypothesis that the induction of the electromagnetic field causes better efficiency in water use can be confirmed. Thus, the plants had superior productive performance, in which they spent less energy on growth and directed it to fruiting [10].

The variables related to the fruit were significantly affected by the nutrient solution strengths. The increase in nutrient solution strength provided a reduction in the diameter, length and number of fruits. Such effects are products of nutritional imbalance [29], and the species preservation process makes the plant seek defense against the stress generated, causing energy to be spent for other purposes [24].

In addition, it can be seen in the present study that the increase in nutrient solution strength provided a lower total weight of fruits with apical rot. According to a study by [30], a lower weight and a greater number of fruits with blossom-end rot are expected. This physiological problem is attributed to a calcium deficiency, which is directly related to toxicity. Excessive application of salts can make the fruits unfit for in natural or processed consumption [31]. The interaction between the factors affected the average fruit diameter, in which doses above 4 dSm^−1^ had no significant effect. The greatest impact was at doses of 1.5 and 2.5 dS m^−1^ when irrigated with ETW water. Thus, it demonstrates that irrigation with ETW impacted the diameter, whereas the dose of 1.5 dmS^−1^ with ETW water presented the largest diameter. The results obtained in the work are in agreement with those cited for tomato [32] potatoes [33], cowpea and eggplant [10,11].

Tomato fruits generally have sufficient acidity to maintain their pH below 4.6 and, for this reason, are considered an acidic typed of food [34]. For the titratable acidity, the results found from the physico-chemical analysis are expected for the tomato culture, which is from 0.3 to 0.4 g 100 g^−1^ [35,36,37]. There is an inverse relationship between titratable acidity and pH, where the higher the acidity, the lower the pH [38] and vice versa. Interestingly, this behavior is observed in this work. It is noteworthy to mention that [38,39] also found, in their studies, that different concentrations of fertigation did not interfere with the pH of the fruit, and the average values obtained were 4.98 and 5.01, respectively. These, data corroborate with the results obtained in this study.

The soluble solids content is of great economic importance in tomato processing. Even a small increase in this quality attribute can significantly increase the industrial yield and reduce the cost of pulp dehydration [40]. This content is directly associated with industrial yield, where each BRIX increase in the raw material has, on average, a 10 to 20% increase in yield [41]. Studying tomato varieties for processing, ref. [42] found that fertilization with 200, 400 and 600 kg ha^−1^ of K_2_O increased the content of soluble solids by 3%, 6% and 8%, respectively, compared to the control group (no potassium fertilization). The greater soluble solids content was obtained in the present study with an increase in the strength of the nutrient solution. However, there was no correlation with the potassium content. This increase may be associated with reduced water in the fruit [43] and with an accumulation of solutes, mainly ions and organic molecules typically produced in plants stressed by salt [44]. As a consequence of this, the concentration of soluble solids in the pulp tends to increase.

The ratio for the tomato crop has indexes within the acceptable range (palatability considered ‘tasty’) with values above 10, as referred by [38]. In the present study, it was possible to verify that, for all treatments, the values were higher than previously mentioned, thus demonstrating that the fruits have the potential for processing [45]. In the work, their evaluation, showed that there was an effect similar to the results observed in the present study.

The interaction of factors significantly affected the physico-chemical characterization parameters. The tomato has a high added value, so the quality of the parameters is fundamental for commercialization. Thus, in the present work, we observed that the nutrient solution strengths and the type of water adopted for irrigation were higher. So, the treatments with a salinity of 5.5 and 7.0 dS m^−1^ with ETW irrigation showed the highest soluble solids, ratio and lowest pH values, thus provoking a better future quality. Such results were also observed by [10,11,12,33,46,47].

In general, the effects of electromagnetically treated water on nutrient solution strength stress were positive, since improvement aspects in fruit quality were verified. The positive effect of electromagnetic water on tomato plants was studied in seedlings under saline stress [46,48], in the germination phase under saline stress [47], and in association with drainage water agriculture [48].

Table 1 presented the nutrient content values in tomato fruits, in which we observed N, K, and S, and there was no significant effect for the interaction. Among them, only S had an impact on the type of water.

We should highlight the Ca content in the fruits in which the ETW irrigation provided the highest concentration, directly related to non-commercial fruits, since Ca is a fundamental element for the non-appearance of blossom-end rot on tomatoes [49,50]. From the best of our knowledge, there is no other work in the literature studying the effects of treatment with ETW that caused this reduction.

## 5. Conclusions

The fertigation doses significantly affected the tomato crop, which can be seen in reduced production, reduced fruit size and an increased number of fruits with apical rot. Therefore, the electromagnetic water treatment increased the fruit quality parameters, mainly in the highest applied doses.

## Figures and Tables

**Table 1:** The concentration of fertilizers diluted in water for fertigation doses.

	**Plant Development Phase**				
**Nutrients (g L^−1^)**	**1.5**	**2.5**	**4.0**	**5.5**	**7.0**
	**dSm^−1^**				
Calcium nitrate	0.6300	1.0500	1.6800	2.3100	2.9400
Urea	0.0600	0.1000	0.1600	0.2200	0.2800
Potassium nitrate	0.2400	0.4000	0.6400	0.8800	1.1200
Potassium chloride	0.1800	0.3000	0.4800	0.6600	0.8400
Potassium sulfate	0.0600	0.1000	0.1600	0.2200	0.2800
Magnesium nitrate	0.1200	0.2000	0.3200	0.4400	0.5600
Magnesium sulfate	0.0600	0.1000	0.1600	0.2200	0.2800
MKP	0.1680	0.2800	0.4480	0.6160	0.7840
Zinc sulfate	0.0018	0.0030	0.0048	0.0066	0.0084
Boric acid	0.0048	0.0080	0.0128	0.0176	0.0224
Chelated iron	0.0120	0.0200	0.0320	0.0440	0.0560
	**Production Phase**				
**Nutrients (g L^−1^)**	**1.5**	**2.5**	**4.0**	**5.5**	**7.0**
	**dSm^−1^**				
Calcium nitrate	0.6684	1.1140	1.7824	2.4508	3.1192
Urea	0.0660	0.1100	0.1760	0.2420	0.3080
Potassium nitrate	0.2580	0.4300	0.6880	0.9460	1.2040
Potassium chloride	0.0180	0.0300	0.0480	0.0660	0.0840
Potassium sulfate	0.0660	0.1100	0.1760	0.2420	0.3080
Magnesium nitrate	0.1320	0.2200	0.3520	0.4840	0.6160
Magnesium sulfate	0.0660	0.1100	0.1760	0.2420	0.3080
MKP	0.1800	0.3000	0.4800	0.6600	0.8400
Zinc sulfate	0.0020	0.0033	0.0053	0.0073	0.0092
Boric acid	0.0054	0.0090	0.0144	0.0198	0.0252
Chelated iron	0.0132	0.0220	0.0352	0.0484	0.0616

**Table 2:** *p*-values are associated with the factors of water types, nutrient solution strengths, the distance between nodes, and biometric and chemical evaluations of the fruits and nutrition.

	**Distance between the Nodes**					
**Factor**	**Distance between Node**	**Distance between 1st and 2nd Node**	**Distance between 2nd and 3rd Node**	**Distance between 3rd and 4th Node**	**Distance between 4th and 5th Node**	**Distance between 5th and 6th Node**
**N.S.S.**	0.0020	<0.0001	<0.0001	0.6380	0.2350	0.0874
**T.W.**	<0.0001	0.5320	0.0548	<0.0001	<0.0001	<0.0001
**T.W. X N.S.S.**	<0.0001	0.2310	0.4120	0.0985	0.4680	0.1230
**C.V. (%)**	8.98	10.87	18.95	12.58	7.58	15.85
	**Biometric rating of the fruits**					
**Factor**	**Branch Weight**	**Average fruit diameter**	**Number of fruits**	**Diameter of fruits**	**Weight non-commercial fruits**	**Average weight**
**N.S.S.**	<0.0001	<0.0001	<0.0001	<0.0001	<0.0001	<0.0001
**T.W.**	<0.0001	<0.0001	<0.0001	<0.0001	<0.0001	0.2360
**T.W. X N.S.S.**	<0.0001	<0.0001	0.0845	0.5963	0.2580	0.0850
**C.V. (%)**	16.78	15.96	18.95	11.36	18.63	9.85
	**Chemical rating**					
**Factor**	**Titratable Acidity**	**SS**	**pH**	**Ratio**		
**N.S.S.**	0.0002	<0.0001	<0.0001	0.0014		
**T.W.**	0.0033	<0.0001	0.0328	<0.0001		
**T.W. X N.S.S.**	<0.0001	<0.0001	<0.0001	<0.0001		
**C.V. (%)**	6.30	2.69	8.99	6.68		
	**Nutritional rating of the fruits**					
**Factor**	**N**	**P**	**K**	**Ca**	**Mg**	**S**
**N.S.S.**	0.0002	0.0049	0.0003	<0.0001	<0.0001	0.0024
**T.W.**	0.1490	0.0427	0.4890	<0.0001	0.0037	0.0008
**T.W. X N.S.S.**	0.1020	0.0030	0.0560	<0.0001	<0.0001	0.8750
**C.V. (%)**	9.87	17.58	7.85	4.39	15.65	8.42
	**Chemical rating**					
**Factor**	**B**	**Cu**	**Fe**	**Mn**	**Zn**	
**N.S.S.**	0.0554	<0.0001	<0.0001	<0.0001	<0.0001	
**T.W.**	<0.0001	0.0370	0.0770	0.0770	<0.0001	
**T.W. X N.S.S.**	0.0060	<0.0001	<0.0001	0.2880	<0.0001	
**C.V. (%)**	10.06	8.28	8.03	9.90	9.43	

Notes: C.V.: Coefficient of variation, T.W.: Type of water; N.S.S.: Nutrient solution strengths.

**Table 3:** Nutrient contents in tomato fruit in response to electrical conductivity application with electromagnetically treated water (ETW) and untreated water (UTW).

**Treatments**		**N**	**P**	**K**	**Ca**	**Mg**	**S**	**B**	**Mn**	**Fe**	**Cu**	**Zn**
		**g kg^−1^**						**mg kg^−1^**				
**Type of water**	ETW	21.56 a	5.19 a	32.60 a	2.17 a	1.48 a	2.45 a	19.52 a	20.33 a	57.26 a	14.70 a	42.70 b
	UTW	20.42 a	4.51 b	31.95 a	1.98 b	1.22 b	2.17 b	14.38 b	19.00 b	54.20 a	15.30 a	49.00 a
**1.5 dSm^−1^**	ETW	18.66	4.21 Ba	33.75	2.85 Aa	1.35 Ba	2.34	17.49 Ba	30.00 Aa	66.00 Aa	13.50 Ba	54.00 Aa
	UTW	18.73	3.97 Ba	30.45	1.60 Bb	1.20 Aba	1.96	14.01 Ab	19.50 Abb	50.50 Bb	14.00 Ba	38.50 CDb
**2.5 dSm^−1^**	ETW	16.87	7.30 Aa	31.70	2.12 Ba	2.70 Aa	2.57	23.86 Aa	21.17 Ba	31.95 Cb	21.17 Ba	33.00 Bb
	UTW	18.41	3.92 Bba	29.65	2.15 Aa	1.10 Bb	2.28	13.85 Ab	14.00 Cb	53.50 Ba	15.50 Ab	78.50 Aa
**4.0 dSm^−1^**	ETW	21.98	5.12 Ba	30.03	2.10 Ba	1.05 Ba	2.56	19.84 Aba	15.00 Bb	65.00 Aa	13.00 Ba	40.50 Ba
	UTW	21.28	4.80 Aba	31.75	2.20 Aa	1.10 Ba	2.40	13.36 Ab	22.50 Aa	48.50 Bb	14.50 Ba	46.00 Bca
**5.5 dSm^−1^**	ETW	24.29	4.99 Ba	35.70	1.95 BCb	1.15 Bb	2.58	18.87 Ba	15.50 Cb	76.00 Aa	12.50 Bb	43.00 Bb
	UTW	22.96	6.31 Aa	39.90	2.15 Aa	1.70 Aa	2.33	15.38 Ab	23.00 Aa	66.00 Ab	20.50 Aba	51.50 Ba
**7.0 dSm^−1^**	ETW	25.99	4.35 Ba	31.83	1.87 Ca	1.13 Ba	2.22	17.54 Ba	20.00 Ca	47.33 Ba	13.33 Ba	43.00 Ba
	UTW	20.72	3.56 Ba	28.00	1.80 Bb	1.00 Ba	1.87	15.30 Ab	16.00 Bca	52.50 Ba	12.00 Ba	30.50 Db
**C.V. (%)**		9.87	17.58	7.85	4.39	15.65	8.42	10.06	9.90	8.03	9.90	9.43
**T.W.**		2.27 ^ns^	5.40 *	0.50	35.05 **	11.10 **	16.07 **	68.03 **	5.02 *	5.02 *	1.22 ^ns^	15.91 *
**N.S.S.**		10.30 **	4.76 *	9.48 *	16.31 **	15.82 **	6.29 **	2.83 ^ns^	19.19 **	19.19 **	14.90 **	15.21 *
**T.W. X N.S.S.**		2.27 ^ns^	5.99 *	0.05 ^ns^	63.97 **	21.69 **	0.30 ^ns^	5.08 **	39.71 **	39.71 **	16.75 **	47.51 **

Abbreviations: ns: Not significant, ** significant at 5%; * significant at 1%; Means followed by the same lower case letters are not significantly different within each cultivar and the same upper case letters are not different for each stre of nutrient solution, according to the Tukey test (*p* ≤ 0.05); C.V.: Coefficient of variation, T.W.: Type of water, N.S.S.: Nutrient solution strengths.

## Data Availability

Not applicable.
